# Supplementary material for: Cd59 and inflammation regulate Schwann cell development
Source: eLife. 2022 Jun 24;11:e76640. doi: 10.7554/eLife.76640 (PMC9232220; doi:10.7554/eLife.76640)
Supplement: Figure 3—figure supplement 1—source data 1. — Unlabeled and labeled images of gel electrophoresis showing wildtype (357 bp) and cd59uva48 (variable transcript size) RT-PCR products at 72 hours post fertilization (hpf). RT-PCR products were compared to 100 bp DNA. [file elife-76640-fig3-figsupp1-data1.pdf]

Source Data for Figure 3 - Figure Supplement 1A

Unlabled, Uncropped Gel

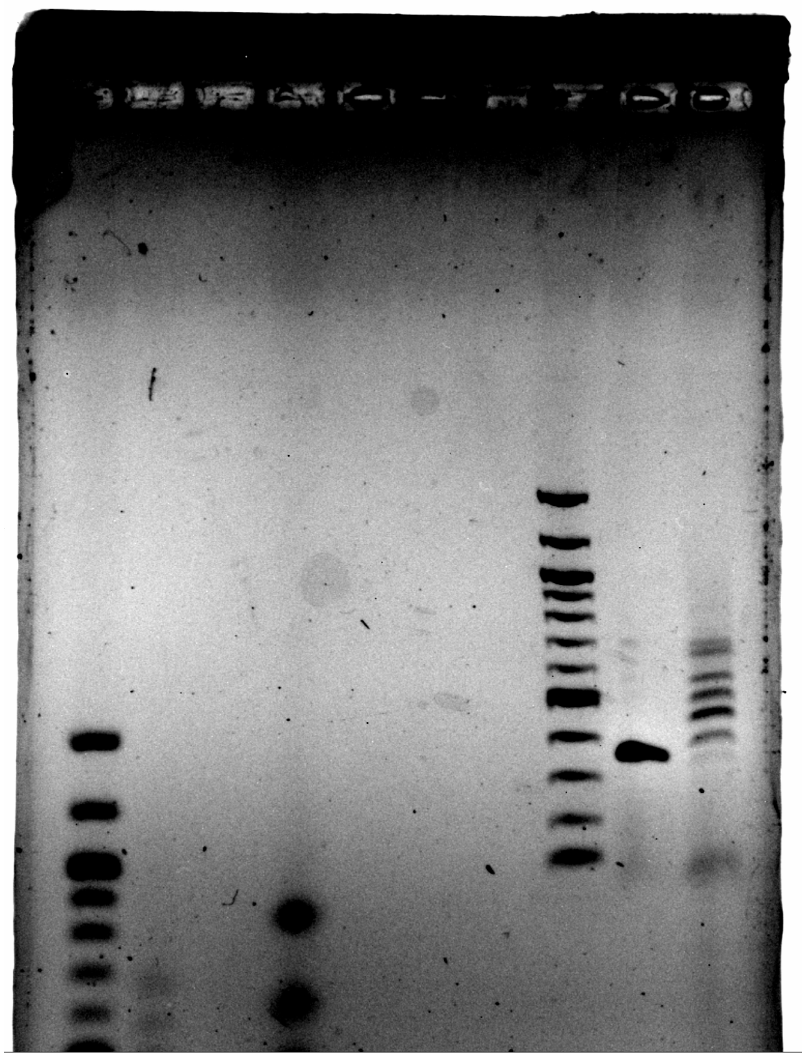

Labeled, Uncropped Gel

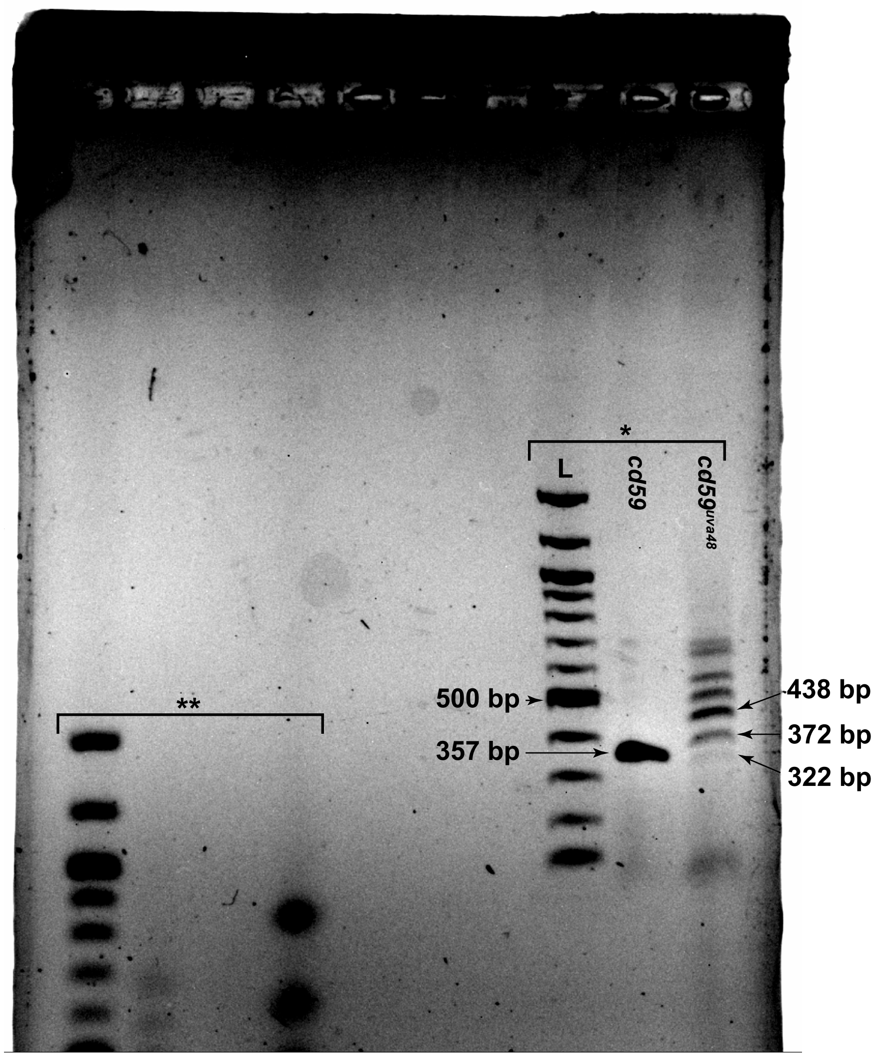

*Data represented in final figure.

**Bands leftover from a different experiment.
