# Supplementary material for: Cd59 and inflammation regulate Schwann cell development
Source: eLife. 2022 Jun 24;11:e76640. doi: 10.7554/eLife.76640 (PMC9232220; doi:10.7554/eLife.76640)
Supplement: Figure 3—figure supplement 1—source data 2. — Unlabeled and labeled images of gel electrophoresis showing wildtype (357 bp) and cd59uva47 (351 bp) RT-PCR products at 72 hours post fertilization (hpf). RT-PCR products were compared to 100 bp DNA. [file elife-76640-fig3-figsupp1-data2.pdf]

Source Data for Figure 3 - Figure Supplement 1B

Unlabled, Uncropped Gel

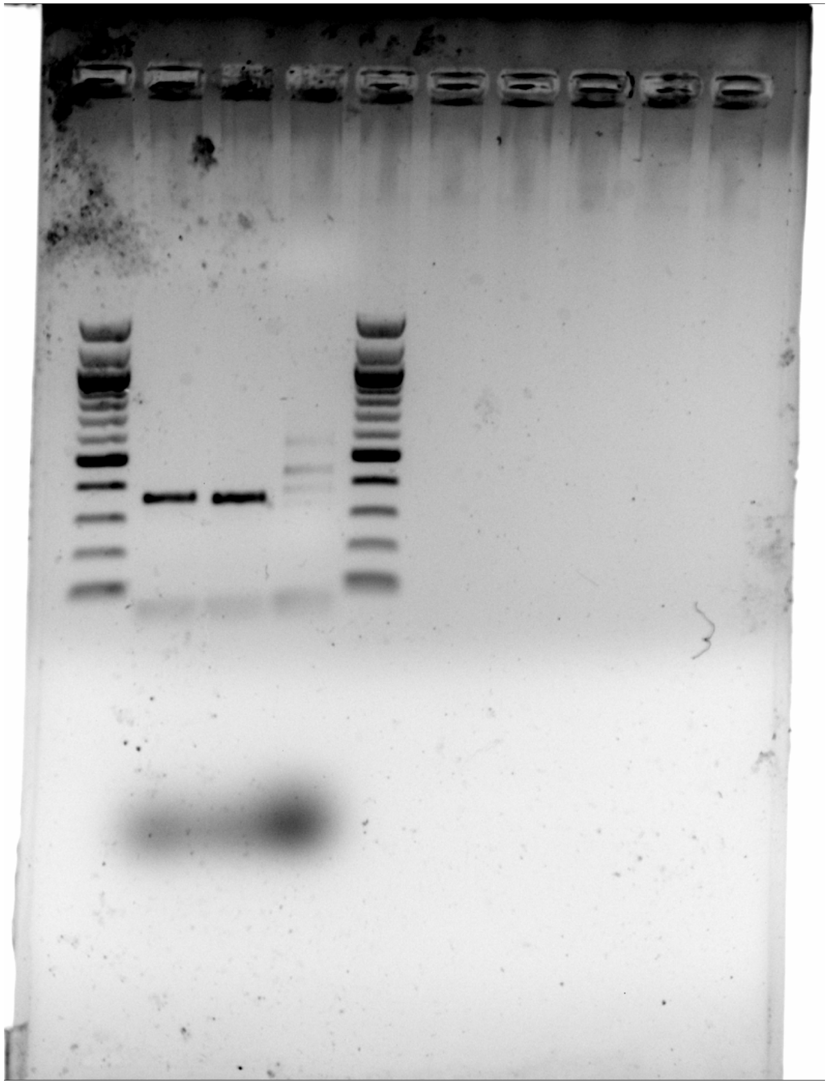

Labeled, Uncropped Gel

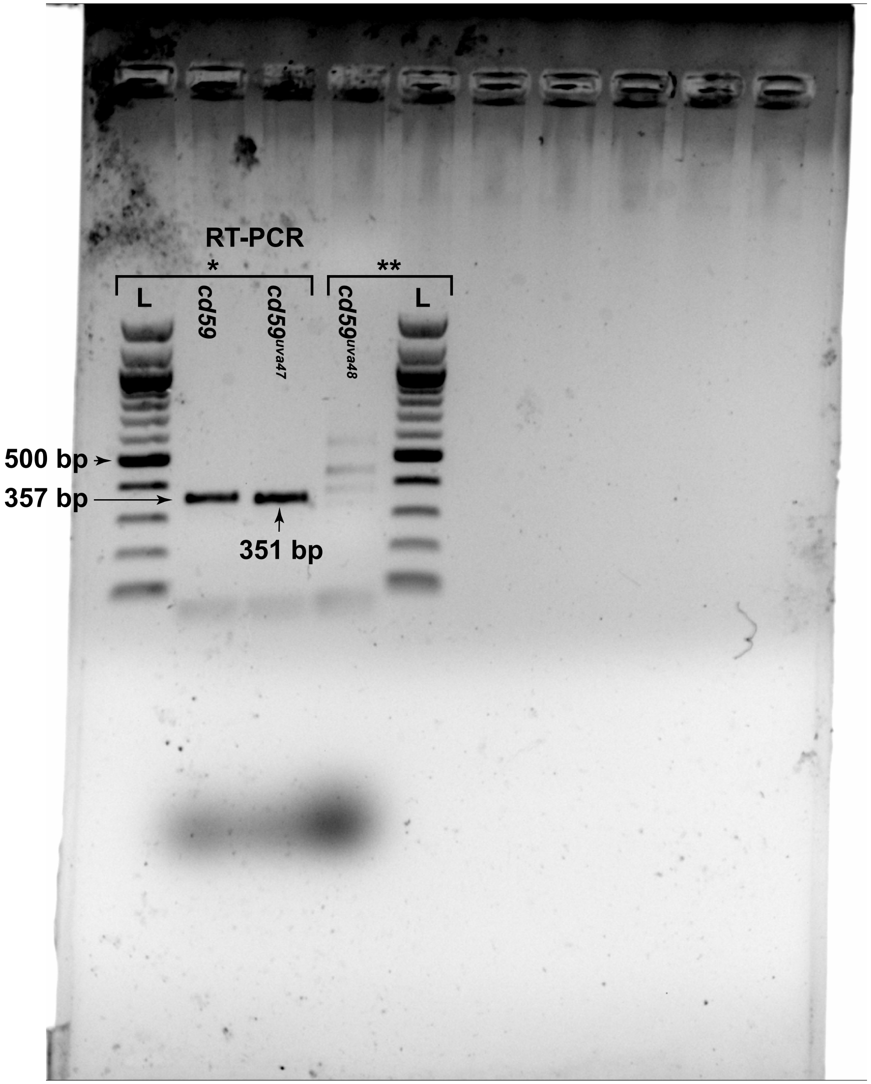

***Data represented in final figure.**

****For author's reference at the time of experiment. Not included in final figure.**
